# Biologic agents in juvenile spondyloarthropathies

**DOI:** 10.1186/s12969-016-0076-6

**Published:** 2016-03-12

**Authors:** María Martha Katsicas, Ricardo Russo

**Affiliations:** Service of Immunology & Rheumatology, Hospital de Pediatría Juan P. Garrahan, Combate de los Pozos 1881, 1245 Buenos Aires, Argentina

**Keywords:** Biologic agents, Juvenile, Spondyloarthropaties

## Abstract

The juvenile spondyloarthropathies (JSpA) are a group of related rheumatic diseases characterized by involvement of peripheral large joints, axial joints, and entheses (enthesitis) that begin in the early years of life (prior to 16^th^ birthday).

The nomenclature and concept of spondyloarthropathies has changed during the last few decades. Although there is not any specific classification of JSpA, diseases under the spondyloarthropathy nomenclature umbrella in the younger patients include: the seronegative enthesitis and arthropathy (SEA) syndrome, juvenile ankylosing spondylitis, reactive arthritis, and inflammatory bowel disease-associated arthritis. Moreover, the ILAR criteria for Juvenile Idiopathic Arthritis includes two categories closely related to spondyloarthritis: Enthesitis-related arthritis and psoriatic arthritis.

We review the pathophysiology and the use of biological agents in JSpA. JSpA are idiopathic inflammatory diseases driven by an altered balance in the proinflammatory cytokines. There is ample evidence on the role of tumor necrosis factor (TNF) and interleukin-17 in the physiopathology of these entities. Several non-biologic and biologic agents have been used with conflicting results in the treatment of these complex diseases. The efficacy and safety of anti-TNF agents, such as etanercept, infliximab and adalimumab, have been analysed in controlled and uncontrolled trials, usually showing satisfactory outcomes. Other biologic agents, such as abatacept, tocilizumab and rituximab, have been insufficiently studied and their role in the therapy of SpA is uncertain. Interleukin-17-blocking agents are promising alternatives for the treatment of JSpA patients in the near future. Recommendations for the treatment of patients with JSpA have recently been proposed and are discussed in the present review.

## Background

**Fig. 1:**
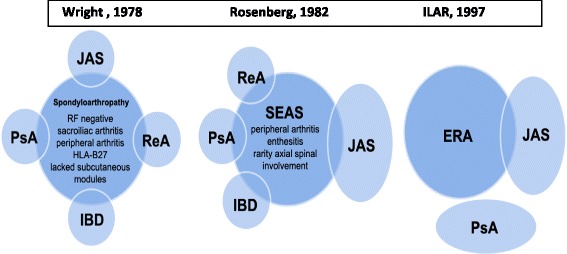
Historical Juvenile Spondyloarthropathy Concept. References: JAS: Juvenile Ankylosing spondylitis; PsA: Psoriatic arthritis; ReA: Recative arthritis; IBD:inflammatory bowel disease. SEAS:Seronegative enthesitis and arthritis syndrome; ERA (ILAR Criteria) Enthesitis_related arthritis: arthritis and enthesitis or arthritis or enthesitis with at least two of the following: sacroiliac joint tenderness and/or inflammatory spinal pain; presence of HLA-B27, family history in at least one first-degree relative with medically confirmed HLA-B27 associated disease, anterior uveitis that is usually associated with pain, redness, or photophobia, onset of arthritis in a boy after 6 years of age. Exclusions: psoriasis confirmed by a dermatologist in at least one first- degree relative, presence of systemic arthritis

The juvenile spondyloarthropathies (JSpA) are a group of related rheumatic diseases characterized by involvement of peripheral large joints, sacroiliitis / spondylitis and enthesitis that begin in the early years of life (prior to 16^th^ birthday) [1]. Spondyloarthropathies are strongly associated with the human leukocyte antigen (HLA) B27. The nomenclature and concept of spondyloarthropathies has changed during the last several decades. The classification criteria defined by the European Spondylarthropathy Study Group (ESSG) marked an important milestone in the common nomenclature for these diseases [2]. Diseases under the spondyloarthropathy nomenclature umbrella in the younger patients include: the seronegative enthesitis and arthropathy (SEA) syndrome, juvenile ankylosing spondylitis (JAS), reactive arthritis, and inflammatory bowel disease-associated arthritis. Enthesitis-related arthritis (ERA) and psoriatic arthritis (PsA) are categories of juvenile idiopathic arthritis (JIA) according to the ILAR classification. The definition of ERA includes children with arthritis and enthesitis, or arthritis plus other features associated with spondyloarthropathy [3–7]. ESSG criteria were developed for adult population and subsequently validated in children. ERA and psoriatic arthritis patients –according to ILAR- might fulfill ESSG criteria [3–8]. ERA represents nearly 15–20 % of patients with JIA in cohort studies, but it does differ in different parts of the world [8, 9]. Figure 1 Probably differences in prevalence could be related with: ethnicity, environment and different microbiological epidemiologies. JSpA are pathogenetically different from other types of JIA [10].

JSpA can be divided into classified and unclassified. Classified diseases includes those that fulfill criteria, but others with peripheral arthritis and presence of HLA-B27 are often considered as unclassified JSpA. Usually those patients with positive HLA-B27 will develop axial involvement later.

JSpA is a group of several conditions, the course of which depends partially upon the presence or absence of the HLA-B27 allele, which predisposes the patient towards a chronic, progressive axial involvement (including sacroiliitis) and final fulfillment of the ankylosing spondylitis (AS) (modified New York) criteria [11]. Other risk factors for development of sacroiliitis include number of active joints (multiple joints involvement) and entheses, hip arthritis, and elevated erythrocyte sedimentation rate (ESR) at disease onset [12, 13]. The development and severity of AS would be linked to certain variables such as: poor efficacy of NSAIDs, elevated erythrocyte sedimentation rate, limitation in range of motion of the lumbar spine, oligoarthritis (defined as four or fewer affected joints), isolated hip arthritis, dactylitis, and onset before the 16^th^ birthday [14–16].

The beginning of treatment for JSpA includes NSAIDs and physiotherapy, but this therapy may not be effective in a large proportion of patients who will require disease-modifying anti-rheumatic drugs (DMARDs). The purpose of this paper is to review the pathogenesis and role of biologic therapy in JSpA.

### Pathophysiology of spondyloarthropathies

Different pathogenetic mechanisms have been proposed for the SpA.

**Fig. 2:**
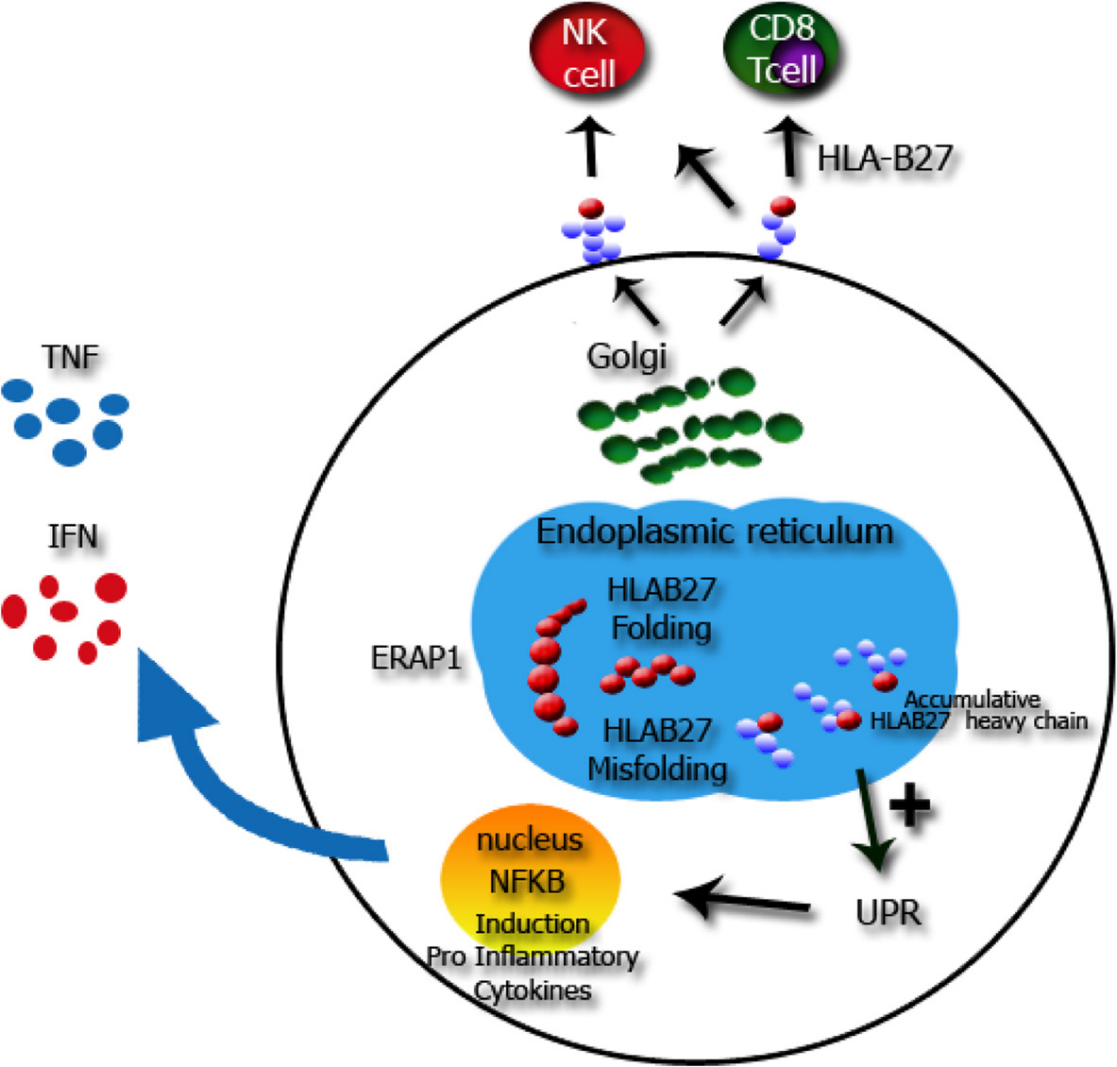
Interactions between genes products and cytokines. References: ERAP1: endoplasmic reticulum aminopeptidasa. UPR : unfolded protein. The misfolding HLA-B27 mechanism in the endoplasmic reticulum, up regulate the UPR. Higher levels of UPR generates an inappropriate genes activation that perpetuate the inflammatory state

The strong association between SpA and HLA-B27 suggests that a genetically determined mechanism is involved in its pathophysiology. The role of HLA-B27 in the pathogenesis of spondyloarthropathies has been extensively studied. The tendency of HLA-B27 heavy chains to misfold in the endoplasmic reticulum would lead to an unfolded protein response and inappropriate cytokine secretion. This hypothesis and the demonstration of the association between some innate immunity-related genes or cluster of genes (such as interleukin IL-1 or ARTS-1 gene [also known as Endoplasmic Reticulum Aminopeptidase or ERAP-1]) with AS support the concept of SpA being a disease with both autoimmune and autoinflammatory mechanisms [17, 18] (Fig. 2). The spondyloarthropathies are a polygenic disease in which polymorphisms in genes related to the innate immune system are involved: *CARD9*, *TNF receptor family member 1A*, *TNF receptor superfamily 15* (*TNFSF15*), *IL-1* (*IL1A*, *ILR2*), *IL-23/IL-17* (*IL-23R*), signal transducer and activator of transcription 3 (*STAT3*) [19].

Molecular mimicry between arthritogenic bacteria and certain domains of the HLA-B27 molecule has been proposed as another probable pathogenic mechanism [20]. An alternative hypothesis focuses on the presentation of certain arthritogenic peptides in the HLA-B27 pocket to the lymphocyte bound CD4 molecule. In this model, CD4+ T cells recognize these bacterial peptides and produce high levels of interferon (INF) γ and other cytokines which act on macrophages. The relationship between HLA- B27 and CD8 + T cells would elicit cross-reactivity between HLA and certain microbial epitopes [21].

Certain cytokines appear to have a prominent role in the pathophysiology of SpA. TNF-α, a potent pro-inflammatory cytokine that shows a pivotal role in inflammatory arthritis [20, 22], has been linked to the inflammation of sacroiliac joints in early AS [23]. Higher serum TNF-receptor (TNFR) 1 and TNFR2 levels have been reported in patients with AS and RA. Serum TNFR1 has been proposed as an inflammatory marker in AS since its serum levels drop in patients on anti-TNF treatment [24].

Recent evidence has implicated the Th23-17 axis in the pathogenesis of SpA [25]. Misfolded HLA-B27 results in the production of IL-23 which induces Th17 cells to release the pro-inflammatory cytokine IL-17 [26]. This cytokine can stimulate different cell types, including synovial fibroblasts, macrophages, and synovial lining cells to produce pro-inflammatory cytokines (such as TNFα and IL-6, Receptor Activator for Nuclear Factor κB Ligand (RANKL), and granulocyte-macrophage colony-stimulating factor (GMCSF), which can increase osteoclast numbers and enhance their activity. Several studies have explored the relationship between IL-17 and IL-23 in SpA [27–30]. Elevated levels of IL-17 in synovial fluid have been also described in 43 patients with ERA by Agarwal et al. [31]. Biologic fluid IL-17 levels were similar in children with ERA and adults with SpA and its concentration was higher in the synovial fluid than in the serum. This study also analyzed the production of IL−6, IL−8, Matrix Metalloproteinase (MMP)-1, MMP-3 and tissue inhibitor of metalloproteinases (TIMP) in supernatants from cultures of fibroblasts derived from synovial cells that were stimulated with IL-17 and TNFα. The results suggest that levels of synovial IL-17 in ERA correlate with disease activity, possibly due to locally induced MMP production by fibroblasts. Synovial fluid IL-17 correlated with number of swollen joints (*r* = 0,35; *p* = 0,05) and number of tender joints (*r* = 0.46; *p* = 0.01); no correlation was found with erythrocyte sedimentation rate [31]. Finally, experimental studies have shown that serum IL-6 levels are elevated in adult patients with AS and PsA [32]. Although these conditions are not pediatric, these studies are the unique evidence of the relationship between IL-6 levels and disease activity in SpA. In addition, IL-6 was expressed in biopsies of sacroiliac joints from patients with AS, especially in those with recent-onset disease [33].

### Biologic agents in JSpA

Over the last decade, biologic agents have demonstrated an impressive beneficial effect on the inflammatory features of several rheumatic inflammatory diseases, including the SpA. Most strikingly, TNF inhibitors have demonstrated a dramatic impact on the symptoms and disease course of adult patients with SpA [34]. However, TNF inhibitors may not be effective for the suppression of new bone and syndesmophyte formation [35, 36]. As pathogenic clues are being elucidated, new therapeutic targets will appear on the horizon.

### Experience in pediatric cohorts

#### Anti-TNF agents

Uncontrolled and controlled studies have demonstrated TNF inhibition is effective for children with JSpA, either classified as ERA according to the ILAR criteria, JAS according to the New York criteria, or JSpA according to EESG criteria.

Henrickson et al. reported sustained efficacy of etanercept in a cohort of pediatric patients with ERA over 2 years [37]. This study showed reduction in (mean): morning stiffness (baseline: 175 m; last visit: 0 min); active joint count (8; 0.3) and ESR (64 mm/h; 33 mm/h).

Additionally, Tse et al. reported improvement in 10 children with JSpA on etanercept or infliximab followed for 1 year [38]. In another retrospective study, 20 patients with JSpA showed good response to TNF inhibition (either etanercept (ETN), infliximab or adalimumab) when they had been refractory to NSAIDs [39]. In this study remission was achieved in 70 % of patients at 6 months after onset of anti-TNF therapy. During this study, other efficacy variables were evaluated such as spinal pain, hip pain and nocturnal awakening. All of them showed improvement. Otten et al. also assessed the effectiveness and safety of TNF-blocking agents in children with ERA. All patients with ERA in whom a biologic agent was initiated between 1999 and 2010 were selected from the Dutch Arthritis and Biologicals in Children registry. Twenty-two patients with ERA were included. Anti-TNF treatment was effective and safe. However, a sustained disease–free state could not be achieved, and no patient could successfully discontinue the TNF-inhibiting therapy [40].

Horneff et al. demonstrated a significant superiority of adalimumab compared with placebo in the treatment of JAS in a double-blind controlled study [41]. An open-label study provided evidence that ETN at 0.8 mg/kg once weekly was effective and well tolerated in pediatric patients with ERA over 12 weeks of treatment. Primary endpoint was the percentage of subjects achieving ACR 30 response criteria [42]. Effectiveness of ETN was also evaluated with the following variables: tender entheseal score, back pain, and nocturnal back pain. ETN was well tolerated in this pediatric population for up to 12 weeks. ACR 30 was achieved by 88,6 % of subjects. Limitations in this trial included its open label design and the retrieval of a placebo-control group from a historical database. Burgos Vargas et al. have also showed that adalimumab (ADA) reduced the signs and symptoms at week 12 in patients with ERA, while safety and efficacy were sustained up to 52 weeks [43]. Likewise, Hugle et al., in an observational study showed early clinical remission in 13/16 (83 %) patients. Children on anti-TNF treatment (ETN or infliximab) had sustained response except for those who had hip disease [44].

Recently, Horneff et al. have published the first phase III randomized, double-blind study to assess the efficacy and safety of ETN therapy in children with ERA. In this study the proportion of patients who achieved ACR pediatric 30, 50, 70, 90, and 100 response rates at week 24 were 93, 93, 80, 56 and 54 % respectively during the initial open-label phase. At week 48, there was a 35 % reduction in the relapse risk in the treated patients during the double-blind phase [45].

Although anti-TNF therapy is considered safe, increased risk of tuberculosis has been widely accepted. A preliminary screening to detect latent or tuberculosis infection should be considered previous to start of treatment [46]. The musculoskeletal involvement of histoplasmosis and other fungal infections should also kept in mind.

Table 1 shows different studies on the use of anti-TNFα agents in patients with JSpA.

**Table 1:** Anti-TNFα in JSpA

Source	Drug	JSpA patients (*n*)	Study duration	Outcome^a^	Study design
Henrickson [37]	ETNERCEPT	8	24 months	improvement^b^	Open-label Uncontrolled
Tse [38]	ETANERCEPT	2			
	INFLIXIMAB	8	12 months	improvement	Open-label Uncontrolled
Sulpice [39]		20 (23 treatments)			
	ETANERCEPT	19	12 months	improvement	Retrospective Cohort
	INFLIXIMAB	3			
	ADALIMUMAB	1			
Otten [40]		22 (24 treatments)			
	ETANERCEPT	20			
	INFLIXIMAB	2	24 months	improvement	Multicenter Observational Register
	ADALIMUMAB	2			
Horneff [41]	ADALIMUMAB	17	6 months	improvement	Double-Blind Placebo-controlled
Horneff [42]	ETANERCEPT	122	3 months	improvement	Open-label
					Uncontrolled
Burgos Vargas [43]	ADALIMUMAB	46	12 months	improvement	Double-Blind Placebo-controlled
Hugle B [44]	INFLIXIMAB	10	84 months		Open-label
	ETANERCEPT	6		improvement	Observational
Horneff G [45]	ETANERCEPT	41	12 months	improvement	Double-Blind Placebo- controlled

References:

^a^Outcome measures observed were different: morning stiffness, active joints count, tender enthesal count, ESR, ACRped 30/50/70/90, inactive disease, ASAS 20/40, CHAQ, BASFI

^b^Improvement was defined according to each author's criteria as decrease in morning stiffness, active joints, tender enthesal count and ESR, improvement in functional capacity (CHAQ and/or BASFI). Also tools used in improvement assessment were ACR ped, BASDAI, JADAS 10, ASAS. inactive disease and remission

#### Other biologic agents (non TNFα blockers)

Several agents that have been effective in patients with JIA have not been specifically studied in patients with JSpA. Different studies about non TNFα blockers have been developed in adult patients with SpA and are discussed below.

Abatacept did not prove useful in AS in a 24 week, prospective, open–label, pilot study involving 30 patients with active AS divided into two groups: TNF-inhibitor naive and TNF-inhibitor refractory. Response was evaluated with Assessment of SpondyloArthritis International Society (ASAS) criteria. ASAS40 was reached by 13 % and 0 % of patients respectively [47]

Tocilizumab (TCZ) showed no benefit in clinical outcomes in 99 AS patients compared with placebo in a 12-week randomized trial. Response was evaluated with ASAS20 [48]. Response rates were 37.3 % and 27.5 % in TCZ and placebo arms respectively (*p* = 0.28) [49]. Only a decrease in C-reactive protein levels was observed as a beneficial indicator. Despite the association between increased serum IL-6 levels and disease activity in patients with AS reported in previous studies, IL-6 blockade did not correlate with clinical effectiveness in clinical practice. However, TCZ was beneficial in AS patients in small case studies [50, 51].

Song et al. demostrated that rituximab (RTX) did not seem to be effective in patients with AS that had not responded to anti-TNFα agents, but it had significant efficacy in anti-TNF-naïve patients in an open-label, phase II clinical trial [52].

The efficacy and safety of Secukinumab (an anti-IL17A monoclonal antibody with proven efficacy in psoriasis), was tested in a double -blindtrial in adult patients with AS. Primary outcome was ASAS20 at 16 weeks. Sixty percent of patients achieved improvement which was sustained up to 52 weeks [53].

### Recommendations for the treatment of JSpA

There are currently no specific recommendations for the treatment of JSpA. Available recommendations address the JIA group as a whole despite ERA being a distinct category. On the other hand, recommendations for adult patients with SpA have been developed and might be applicable to patients with JSpA.

The ASAS recommends the use of TNF blockers for patients with high disease activity despite conventional treatment, which includes non-steroid anti-inflammatories and glucocorticoids, and DMARDs such as methotrexate (MTX) and/or sulfazalazine for adult patients with SpA [54]. AS patients have demonstrated poor response to conventional DMARDs, and it is recommended that TNFα inhibitors be used as first line therapy for patients with axial disease [54]. There are no data showing that traditional DMARDs are effective in the axial disease of SpA [55]. Roychowdhury et al. did not show any significant improvement in disease activity as measured by the Bath Ankylosing Spondylitis Disease Activity Index (BASDAI) and CRP in AS patients receiving methotrexate as compared to patients in the placebo group [56]. Other studies have showed sulfasalazine is ineffective in axial disease. A multicenter, double-blind, placebo-controlled trial showed that sulfasalazine is only effective in peripheral arthritis [57]. Recently, the American College of Rheumatology (ACR) has elaborated recommendation for the treatment of Ankylosing Spondylitis that could be used for patients below 18 years of age [58].

Important differences exist in how spondyloarthritis begins and progresses in children and adults, supporting the need for pediatric-specific recommendations. As previously shown, there is evidence that TNFα inhibitors are beneficial in JSpA, consistent with results from multiple adult SpA trials. Moreover, both peripheral arthritis and enthesitis, two important therapeutic targets in JSpA, appear to be responsive to TNFα inhibition [59].

The ACR recommendations for the treatment of JIA do not address JSpA as a distinct category, but provide a dedicated strategy for children with sacroiliitis. It is recommended to use a TNFα inhibitor early for patients with axial involvement who exhibit radiographic damage (defined as erosions or joint space narrowing), and moderate or high disease activity (meeting one or two of the following: erythrocyte sedimentation rate (ESR) or C-reactive protein greater than twice upper limit of normal, physician global assessment of overall disease activity ≥7 of 10 or patient/parent global assessment of overall well-being ≥4 of 10) [60]. The treatment of enthesitis was omitted from the 2011 recommendations due to the lack of sufficient supporting evidence. According to these recommendations, in the presence of low disease activity and lack of radiographic damage, TNFα inhibitors should be used only after MTX or Sulfasalazine have proven ineffective for at least 3 months. In patients with active peripheral arthritis but no active sacroiliitis, treatment with anti-TNF therapy is recommended only after a 3 to 6 months-long MTX treatment has proven to be ineffective [60].

Several drawbacks actually limit the acquisition and use of sound evidence for the development of specific recommendations for the treatment of JSpA. Specific disease outcome measures have not been usually used in therapeutic trials performed in a pediatric population. Moreover, assessment of enthesitis has seldom been included. The Juvenile Spondyloarthtris Disease Activity Index still needs to be prospectively validated in a large international cohorts [61]. Also, the design of prospective trials should include more homogeneous cohorts, larger sample size, longer observation periods, controlled use of concomitant medications, and probably inclusion of patients with early disease. Also, clear definitions of active/inactive disease, flare, and remission in JSpA are needed.

## Conclusions

The spondyloarthropathies are a diverse group of arthritides, classically involving large joints of the lower extremities, axial joints and entheses. There is evidence for a pathogenetic role of pro-inflammatory cytokines, especially TNF-α, in the pathogenesis of JSpA. IL-1, IL-6, and IL-17 may also play a meaningful pahogenetic role.

Anti-TNF agents have proven to be effective and safe for the treatment of JSpA in uncontrolled and controlled trials. Other biologic agents have not been formally tested in JSpA, but could be useful in particular cases.

There are currently no treatment recommendations for JSpA. Evidence does favor the early use of anti-TNF agents in patients with JSpA with active axial involvement or MTX-refractivity. In the coming years, a deeper understanding of the pathogenic mechanisms involved in JSpA may provide scientific evidence for other effective therapies, such as IL-17 inhibitors.
